# Critical Role of Cysteine-Rich Protein 61 in Mediating the Activation of Renal Fibroblasts

**DOI:** 10.3389/fphys.2019.00464

**Published:** 2019-05-03

**Authors:** Hang Liu, Long Zhao, Jisheng Zhang, Chenyu Li, Xuefei Shen, Xuemei Liu, Wei Jiang, Congjuan Luo, Yanfei Wang, Lin Che, Yan Xu

**Affiliations:** ^1^Department of Nephropathy, The Affiliated Hospital of Qingdao University, Qingdao, China; ^2^Key Laboratory, Department of Otolaryngology-Head and Neck Surgery, The Affiliated Hospital of Qingdao University, Qingdao, China

**Keywords:** cysteine-rich protein 61, renal fibrosis, fibroblasts, proliferation, bioinformatics

## Abstract

**Objective:**

To explore the expression of cysteine-rich protein 61 (Cyr61) in ischemic renal fibrosis and the role of Cyr61 in mediating the activation of renal fibroblasts.

**Methods:**

(1) The rat model of renal fibrosis was established after ischemia-reperfusion acute renal injury (IR-AKI). We detected the renal function by biochemical test, evaluated the fibrosis by Masson staining, and detected the expression of Cyr61 by western blotting. (2) Bioinformatics technique was adopted to analyze the expression of Cyr61 in activated renal fibroblasts. (3) Normal rat kidney fibroblast cells (NRK-49F cells) with over-expression of Cyr61 (Cyr61^+^) and low-expression of it (Cyr61^--^) were established by plasmid transfection. Then part of the cells were activated by TGF-β1 and NRK-49F cells were divided into control group, activated group, Cyr61^+^/Cyr61^--^ group and Cyr61^+^/Cyr61^--^ activated group. The expression of Cyr61 and fibrosis related factors (Col1α1, Col3α1, MMP9, and MMP13) were ascertained by PCR and western blotting. Cell proliferation was discovered by CCK8 method, cell cycle was analyzed by flow cytometry, and the transcription of cell senescence related factors (P53, P21, Rb, and P16) were ascertained by PCR method.

**Results:**

(1) In the process of fibrosis after IR-AKI, the area of collagen fiber was most obviously at AKI 1W, while the Cyr61 protein was at the lowest level at AKI 1W. (2) Gene chip analysis showed that the expression of Cyr61 was decreased in renal fibroblasts after IR. (3) Compared with control group, Cyr61^+^ group expressed less Col1α1 or Col3α1, as well as more MMP9 and MMP13. At the same time, the proliferation of Cyr61^+^ group decreased and cells in G1 phases increased with more transcription of P53, P21, and Rb (all *P* < 0.05). Compared with activated group, the results of Cyr61^+^ activated group were similar to the above. The above effects of low expression group were just the opposite. In addition, there was no difference in the transcription of P16 among these groups (*P* > 0.05).

**Conclusion:**

Cyr61 may not only inhibit the fibrotic phenotype of fibroblasts, but may also inhibit proliferation by promoting fibroblasts arrest in G1 phase through the P53/P21/Rb interrelated cell senescence pathway, subsequently affecting the process of ischemic renal fibrosis.

## Introduction

Renal fibrosis is a common pathological manifestation in various chronic renal diseases, of which the essence is excessive extracellular matrix (ECM) deposition in the tubulointerstitial region. Activated renal fibroblasts are the most important ECM secreting cells in the early interstitial fibrosis (Wynn, 2010). The activation of fibroblasts and the deposition of ECM will lead to renal tubules atrophy and eventually to chronic renal disease (Venkatachalam et al., 2010). Ischemia-reperfusion is a major cause of acute kidney injury (AKI), which can promote the synthesis and release of various vasoactive substances and cytokines to active fibroblasts. Once AKI occurs and surpasses body compensation, renal interstitial fibrosis will start. The extent of renal interstitial fibrosis is highly correlated with renal dysfunction. Further, the initial ischemic injury may be profound or even irreversible through the development of renal interstitial fibrosis.

Cysteine-rich protein 61 (Cyr61) is a secretory protein with a variety of biological effects. It was found to be highly expressed at the early stage of IR-AKI tissue, with the peak of expression at 4∼8 h and further decrease to near-normal level in 24 h (Muramatsu et al., 2002; Li et al., 2018). But in the late stage of IR-AKI (after 1 day), the expression level of Cyr61 and the link between Cyr61 and renal fibrosis were unknown. Studies have found that Cyr61 could inhibit the activation of fibroblasts to reduce organ fibrosis in liver (Kim et al., 2013) and skin (Jun and Lau, 2010), while it could induce biliary duct cell hyperplasia to accelerate the repair process of liver injury (Kim et al., 2015). We found that Cyr61 could promote the proliferation of renal tubular epithelial cells (Xu et al., 2014), inhibit endoplasmic reticulum stress to inhibit apoptosis (Rui et al., 2016), and protect renal tubular cells. However, it is not clear whether Cyr61 affects renal fibroblasts in a cell-specific way. Therefore, the aim of this study is to explore the expression of Cyr61 in ischemic renal fibrosis and the role of Cyr61 in mediating the activation of renal fibroblasts.

## Materials and Methods

### Animal Models

Clean, healthy male SD rats were purchased from Jinan Yuepeng Animal Center, weighing approximately 150–180 g. Forty SD rats were adaptively fed for one week and then randomly divided into the following 6 groups: control group, after AKI 1-day group (AKI 1D), after AKI 1-week group (AKI 1W), after AKI 2-week group (AKI 2W), after AKI 4-week group (AKI 4W), and after AKI 8-week group (AKI 8W). After being anesthetized, the right kidneys of all experimental rats were removed, and the left renal pedicles were closed with non-invasive vascular clamp for 40 min and then unclamped. During this process, we observed that the kidney color gradually changed from bright red to dark red, and then rapidly from dark red to bright red after unclamped. The left kidney of the control group was not clipped. We collected 2 ml of blood from the rat heart and removed the left kidneys at different time after the operation. The levels of serum creatinine (Scr) were detected by Olympus AU2700 automatic biochemistry analyzer.

### Histopathological Examination

The kidney tissue was fixed by 10% formalin solution for 18 h, then dehydrated, paraffin embedded, sectioned, and had Masson staining performed on it. Five fields of view were randomly selected in each slice under X400 light microscope, and the area of collagen fiber revealed by Masson staining was counted by Image J software. The relative area was calculated by left kidney/right kidney to exclude individual differences.

### Bioinformatics Analysis

We collected ischemic kidney-related samples in the public gene chip database–NCBI GEO^1^ and downloaded and used the original chip data (TAR of CEL) and platform annotations for data preprocessing and differential gene screening. We used the RNA degradation curve and NUSE box line chart to exclude the unqualified samples (the slope of the RNA degradation curve is significantly larger or the NUSE value is more than 1.05). The samples used for follow-up analysis were standardized by RMA, and the expression of Cyr61 in each group of ischemic reperfusion renal fibroblasts was calculated by linear regression model Limma package (Ritchie et al., 2015).

### Cell Culture, Activation, and Plasmid Transfection

Normal rat kidney fibroblast cells (NRK-49F) cells were purchased from the Laboratory of Science and Education Building, Qingdao University Affiliated Hospital. The cells were treated with 10% fetal bovine serum, 1 × 10^5^ units/L penicillin, and 100 mg/L streptomycin in RPMI-1640 medium. The activation of NRK-49F cells was induced for 48 h by 5 μg/L TGF-β1 (PeproTech, United States), a recognized strong fibrosis factor. NRK-49F cells were divided into control group, overexpression group, and low-expression group by plasmid transfection. Cyr61 silencing plasmids were purchased from JiKai Company, and the target sequence was 5′-CTACAGTCTGTTCAACGAT-3′. For the overexpression vector, Cyr61 was subcloned into PiggyBac (PB) vector. These plasmids were transfected with polyethyleneimine (PEI) reagent (Proteintech, United States) into cells for 48 h following the instructions from the manufacturer. To exclude the interference of plasmids not transferred into cells, the cells were washed with cold PBS twice and then harvested. PCR method can demonstrate the different expression of Cyr61 after transfection.

### Cell Viability Assay

The cells were uniformly inoculated into plates to stably cultivate and grouping. In 96-well plates, the cells were incubated for 2 h with serum-free medium containing 10% reagent of Cell Counting Kit-8 (CCK-8, Dojindo Molecular Technologies, Kumamoto, Japan) at 37°C. The absorbance at 450 nm of every well was measured to detect cell viability by a Bio-Rad Microplate Reader. The control group was normalized to one as the relative absorbance.

### Quantitative RT-PCR (qRT-PCR)

Total cellular RNA was extracted by using Trizol reagent (Invitrogen, United States), and cDNA was obtained by 500 ng RNA referring to the manufacturer’s instructions (Takara Corporation, Japan). The expression level of target mRNA was detected by real-time fluorescence quantitative PCR kit (Takara Corporation, Japan) using FastStart Universal SYBR Green Master (ROX) (Roche, United States). All primers were designed and synthesized by Liuhe Huada Gene Science and Technology Limited Company (Beijing, China): GAPDH(Fw5′-TCGACAGTCAGCCGCATCTT-3′ and Rv:5′-GAGTTAAAAGCAGCCCTGGTG-3′); Cyr61(Fw5′-GCAGTTGGAAAAGGCAGCTC-3′ and Rv:5′-ACAGGTCTTTGAGCACTGGG-3′); Col1α1(Fw5′-CTTTGTGGACCTCCGGCTC-3′ and Rv:5′-TCAGGTTTCCACGTCTCACC-3′); Col3α1(Fw5′-GAAATGGCGACCCTGGTCTT-3′ and Rv:5′-CCATTCCTCCGACTCCAGAC-3′); MMP9(Fw5′-TGGATAACGAGTTCTCTGGCG-3′ and Rv:5′-CCGGTTGTGGAAACTCACAC-3′); MMP13(Fw5′-CATCCCGAGACCTCATGTTCA-3′ and Rv:5′-TCCTCAAAGTGAACCGCAGC-3′); P53(Fw5′-GCGACTACAGTTAGGGGGTA-3′ and Rv:5′-GCTCGATGCTCATATCCGAC-3′); P21(Fw5′-TTGTGATATGTACCAGCCACAG-3′ and Rv:5′-CCATGAGCGCATCGCAATC-3′); Rb(Fw5′-GCGGAGTCCAAATTCCAACAG-3′ and Rv:5′-TGTCCCGAGGGTCTACAGTG-3′); P16(Fw5′-CGTGCGGTATTTGCGGTATC-3′ and Rv:5′-GGCCTAACTTAGCGCTGCTT-3′). The expressions of target mRNA were calculated by the 2^-ΔΔct^ method and normalized to GAPDH expression in each sample.

### Western Blotting

After the renal tissue or cells were fully lysed and the protein was denatured, the protein was quantified by BCA^TM^ Protein Assay Kit (Pierce, Appleton, WI, United States). The same quality protein was separated by sodium dodecyl sulfate-polyacrylamide gel electrophoresis (SDS–PAGE) and transferred to a polyvinylidene fluoride (PVDF, Millipore, Bedford, MA, United States) membrane. The PVDF membrane was blocked with 5% skim milk for 1 h at room temperature and incubated overnight by a primary specific antibody (diluted 1: 2000) at 4°C. Collagen β (Col3) antibody and Matrix metalloproteinase 9 (MMP9) antibody were purchased from American Abcam. Cyr61 antibody and β-actin antibody were purchased from American CST. Then, the secondary antibody was labeled with horseradish peroxidase, incubated for 1 h, and detected using a chemiluminescence kit (Pierce, United States). The western blot bands were detected by the chemiluminescence gel imaging system and scanned with Image J software. The ratio of the target protein to the reference protein was used to correct the error.

### Cell Cycle Assay

Cells in logarithmic growth phase were centrifuged to collect and precooled with 70% ethanol overnight. The cells were washed and resuspended with 4°C PBS solution, and the cell suspension concentration was adjusted to 1 × 10^9^/L. A total of 500 μL propidium iodide staining (PI, Beyotime Biotechnology, China) solution was added and incubated at 4°C for 30 min in the dark for flow cytometry analysis (BD FACS Calibur, United States).

### Statistical Analysis

All data were expressed as means ± standard deviation (SD) and analyzed in GraphPad Prism 5.0 software (GraphPad Software, San Diego, CA, United States) by using one-way analysis of variance (ANOVA) among diverse groups and Student’s test among independent samples when appropriate. A value of *P* < 0.05 was considered statistically significant.

## Results

### Renal Fibrosis and Cyr61 Protein After Ischemic Acute Kidney Injury in Rats

**FIGURE 1:**
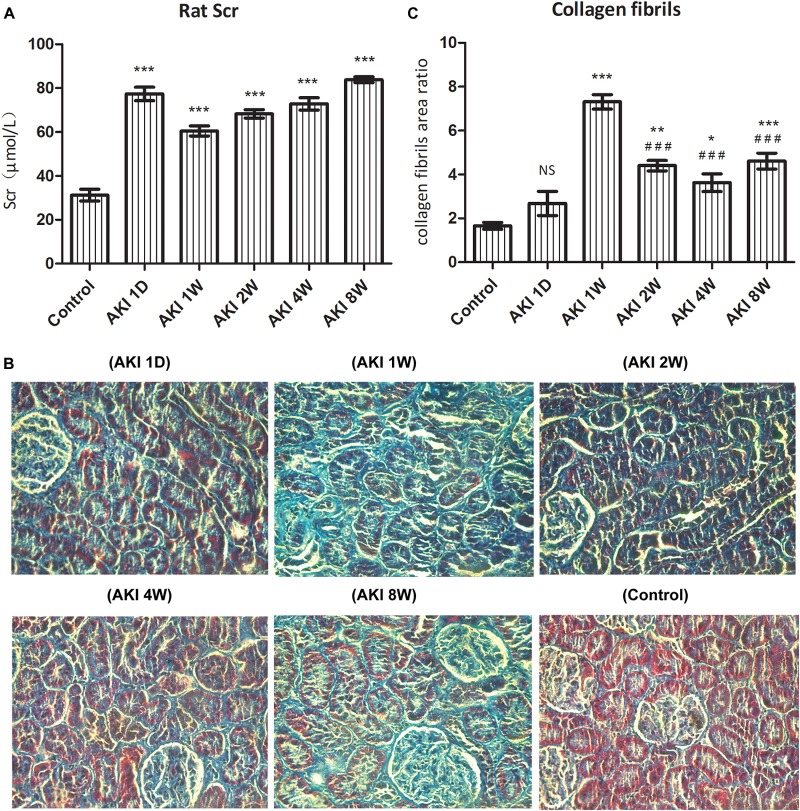
Renal dysfunction and fibrosis after ischemic acute kidney injury in rats. After clamped the right renal pedicle for 40 min, the level of serum creatinine (Scr) was detected by automatic biochemistry analyzer **(A)**. The fibrosis was evaluated by pathological section and Masson staining **(B)**. The relative area of collagen fiber was counted by Image J software **(C)**. NS, no significance, ^∗^*P* < 0.05, ^∗∗^*P* < 0.01, and ^∗∗∗^*P* < 0.001 vs. Control; ^###^*P* < 0.001 vs. AKI 1W.

Scr was increased dramatically, >50% of the baseline value, and reached the level of AKI upon surgery. Scr was increased significantly to more than 2 times at 1 day after IR (*P* < 0.001, Figure 1A), showing a continuous high level after IR-AKI (*P* < 0.001, Figure 1A), which suggested that the renal function is continuously impaired.

In the control group, the structure of renal tubules was clear, and the collagen fibers of the renal interstitium were thin and few. Compared with the control group, the area of collagen fiber was increased significantly at AKI 1W, 2W, 4W, and 8W (*P* < 0.05, Figure 1B,C). The statistical results of Image J software showed that the area of collagen fiber was the largest at AKI 1W (*P* < 0.001, Figure 1C), and the area of AKI 2W, 4W, and 8W collagen fibers decreased significantly compared with the AKI 1W group (*P* < 0.001, Figure 1C).

**FIGURE 2:**
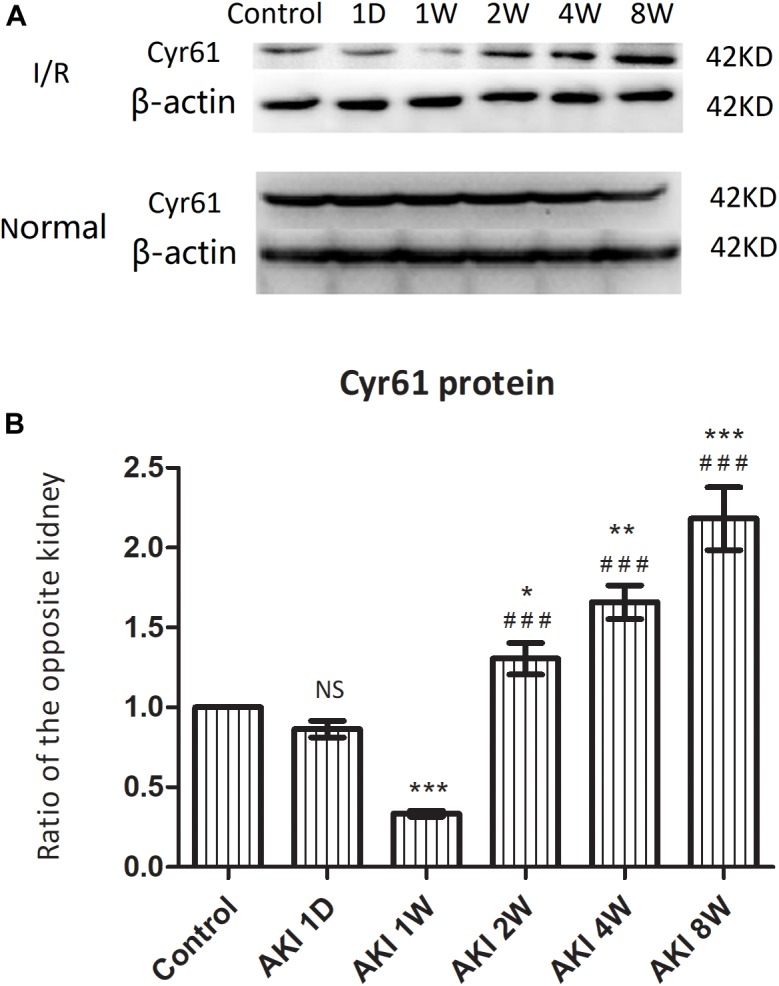
The expression of Cyr61 protein in the fibrosis rat model after IR-AKI. The expression of Cyr61 protein was detected by western blotting **(A)**. Relative protein levels based on western blot results **(B)**. NS, no significance, ^∗^*P* < 0.05, ^∗∗^*P* < 0.01, and ^∗∗∗^*P* < 0.001 vs. Control; ^###^*P* < 0.001 vs. AKI 1W.

Western blotting was used to detect the protein expression of Cyr61 in kidneys after IR-AKI relative to contralateral normal kidneys. Compared with the control group, the expression of Cyr61 was decreased at AKI 1W. Compared with the AKI 1W group, the levels of 2W, 4W, and 8W were increased to varying degrees (*P* < 0.001, Figure 2A,B). These data indicated an opposite trend between Cyr61 protein and renal fibrosis after IR-AKI, suggesting that Cyr61 might interact with renal fibrosis.

### Cyr61 Was Poorly Expressed in Renal Fibroblasts After IR-AKI

**FIGURE 3:**
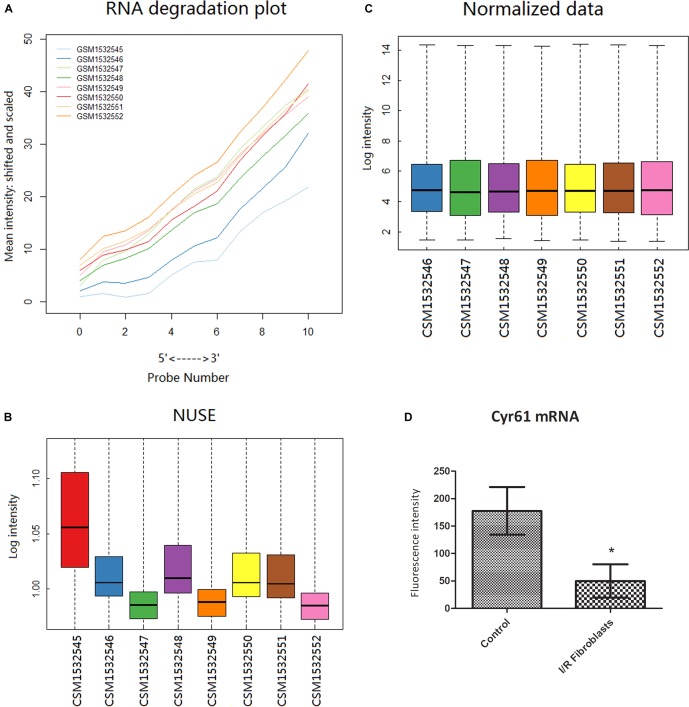
Cyr61 was low expressed in renal fibroblasts at 3 days after IR. Bioinformatics was used to preprocess the GSE62732 gene chip data and calculate the difference of Cyr61 expression. The RNA degradation curve **(A)** and NUSE box plot **(B)** were used to screen unqualified samples (the slope of the RNA degradation curve is significantly larger or the NUSE value is more than 1.05). **(C)** RMA standardization was used for subsequent analysis. **(D)** The expressions of Cyr61 gene in control and IR fibroblasts. ^∗^*P* < 0.05.

From the GEO database GP1261 gene chip platform, 8 samples in GSE62732 chips were obtained, including 3 samples of normal renal fibroblast and 5 renal fibroblast samples at 3 days after IR-AKI. Bioinformatic methods showed that the GSM1532545 chip was not qualified (Figure 3A,B), and the results of data after excluding GSM1532545 showed that the transcription of Cyr61 in the renal fibroblasts decreased significantly at 3 days after IR (*P* < 0.05, Figure 3C,D). These results conformed to the opposite trend between Cyr61 protein and renal fibrosis after IR-AKI and prompted that Cyr61 may act on activated fibroblasts and then affect renal fibrosis.

### Cyr61+ NRK-49F Cells Were Constructed

**FIGURE 4:**
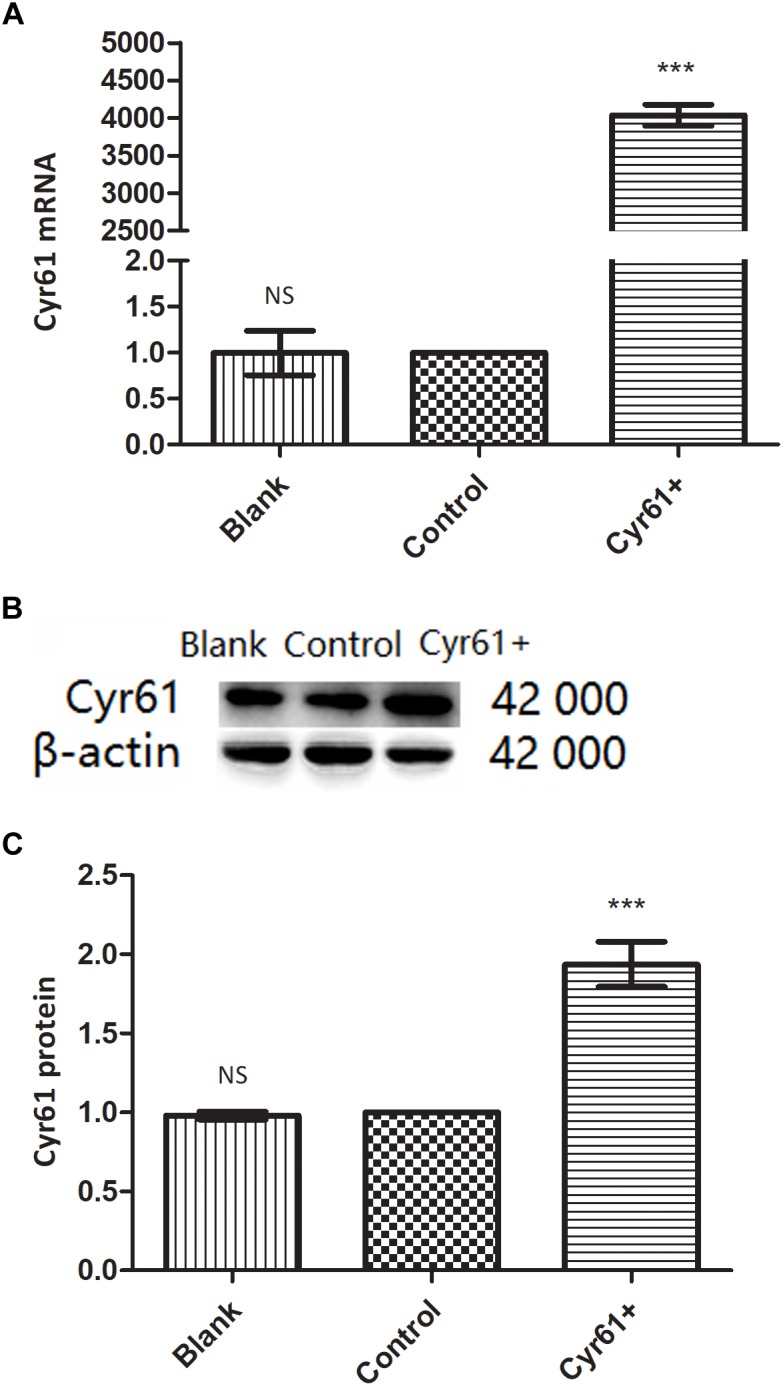
NRK-49F cells with over expression of Cyr61 (Cyr61^+^) were constructed by plasmids transfection. NRK-49F cells were grouped into blank group and control group (null vector transfection) and Cyr61^+^ group (over-expression vector transfection). After transfection for 48 h, the transcriptions of Cyr61 were detected by real time PCR **(A)** and the expression of Cyr61 protein was detected by western blotting **(B)**. Relative protein levels based on western blot results **(C)**. NS, no significance; ^∗∗∗^*P* < 0.001 vs. Control.

To explore the role of Cyr61 on fibroblasts directly, we constructed fibroblasts with Cyr61 overexpression by plasmid transfection. NRK-49F cells were divided into blank group (Blank), control plasmid group (Control), and Cyr61 overexpression plasmid (Cyr61^+^) group. PCR results showed that compared with the Blank and Control groups, the Cyr61 mRNA was higher (about 4000 times) in the Cyr61^+^ group (*P* < 0.001, Figure 4A), but there was no significant difference between the Blank and Control groups (*P* = 0.99, Figure 4A). Furthermore, western blotting results showed that the Cyr61 protein in the Cyr61^+^ group was higher than that of the Blank and Control (*P* < 0.001, Figure 4B,C) groups, but there was no significant difference between the Blank and Control (*P* = 0.50, Figure 4B,C) groups.

### Cyr61 Affected Fibrosis-Related Factors in Renal Fibroblasts

**FIGURE 5:**
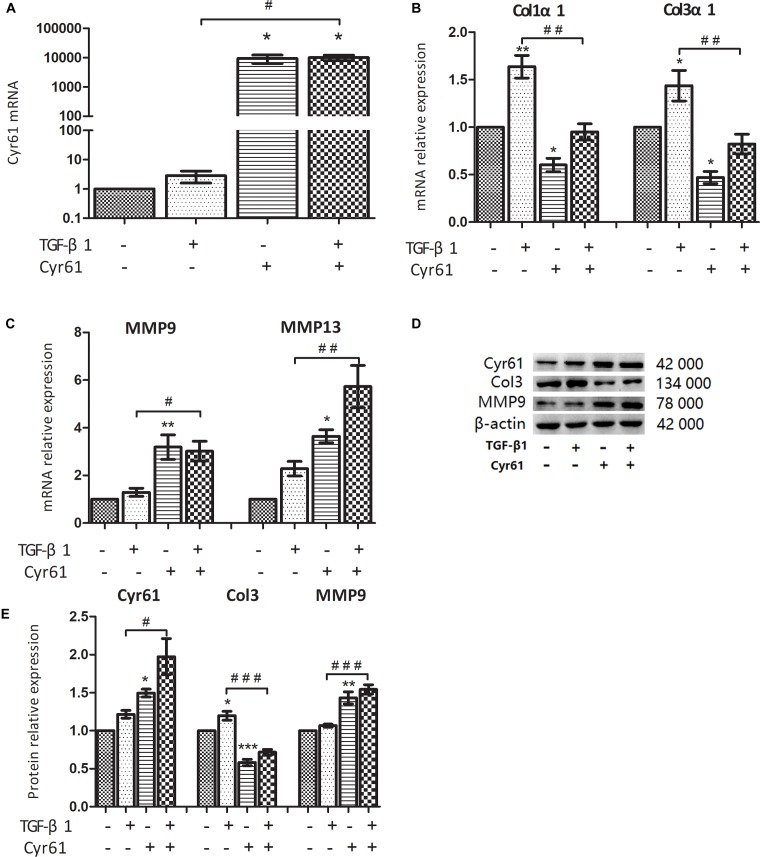
Cyr61 inhibited the fibrosis phenotype of normal and activated renal fibroblasts. NRK-49F cells were grouped into four groups: control group (null vector transfection), activated group (5 μg/L TGF-β1), Cyr61^+^ group (over-expression vector transfection), and Cyr61^+^ activated group (over-expression vector transfection + 5 μg/L TGF-β1). The transcriptions of Cyr61, fibrosis factors (Col1α1 and Col3α1) and anti-fibrosis factors (MMP9 and MMP13) were detected by real time PCR **(A–C)**. The expression of Cyr61, Col3, and MMP9 protein was detected by western blotting **(D)**. Relative protein levels based on western blot results **(E)**. ^∗^*P* < 0.05, ^∗∗^*P* < 0.01, and ^∗∗∗^*P* < 0.001 vs. control group; ^#^*P* < 0.05, ^##^*P* < 0.01, and ^###^*P* < 0.001 vs. activated group.

In this study, 5 μg/L TGF-β1 was used to activate renal fibroblasts, and the cells were divided into control group, activated group, Cyr61^+^ group, and Cyr61^+^ activated group. PCR showed the transcription of Cyr61 in each group, which was extremely high in Cyr61^+^ group and Cyr61^+^ activated group (*P* < 0.05, Figure 5A). Western blotting also showed that Cyr61 protein increased significantly in the Cyr61^+^ group and Cyr61^+^ activated group (*P* < 0.05, Figure 5D,E).

Compared with the control group, the collagen fiber (Col1α1 mRNA, Col3α1 mRNA, and Col3 protein) was decreased, whereas the matrix metalloproteinases with ECM regulation function (MMP9 mRNA, MMP13 mRNA, and MMP9 protein) were increased in the Cyr61^+^ group (all *P* < 0.05, Figure 5B–E). In the activated group, the expression of these collagen fibers was increased (all *P* < 0.05, Figure 5B–E); however, the change of matrix metalloproteinases was not obvious. And compared with the activated group, the expression of these collagen fibers was decreased, whereas the matrix metalloproteinases were increased in the Cyr61^+^ activated group (all *P* < 0.05, Figure 5B–E).

### Cyr61 Inhibited the Viability, Arrested the Cell Cycle and Increased the Transcription of Senescence-Inducing Pathway Related Factors in Renal Fibroblasts

**FIGURE 6:**
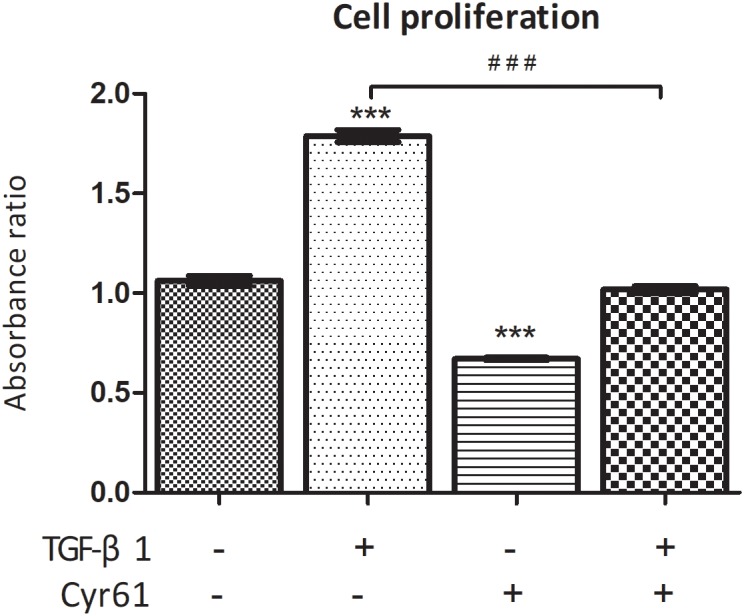
Cyr61 inhibited the viability of normal and activated renal fibroblasts. The cell viability was assessed by CCK-8 assay. ^∗∗∗^*P* < 0.001 vs. control group; ^###^*P* < 0.001 vs. activated group.

Compared with the control group, the cell proliferation of activated group was obviously enhanced, whereas it was obviously weakened for the Cyr61+ group (*P* < 0.001, Figure 6). However, compared with activated group, the proliferation of Cyr61^+^ activated group was significantly decreased (*P* < 0.001, Figure 6).

**FIGURE 7:**
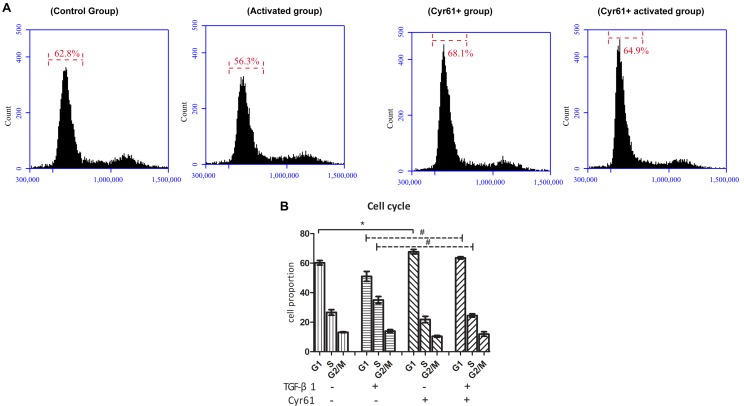
Cyr61 arrested the cell cycle of renal fibroblasts. NRK-49F cells were divided into control group, activated group, Cyr61^+^ group, and Cyr61^+^ activated group. The cell cycle was detected by flow cytometry **(A)**. Percentage of all phases based on cell cycle results **(B)**. ^∗^*P* < 0.05 vs. control group; ^#^*P* < 0.05 vs. activated group.

At the same time, the proportion of cells in the G1 phase of Cyr61^+^ group was increased, whereas the proportion of the activated group was decreased compared to the control group (*P* < 0.05, Figure 7A,B). Compared with the activated group, the G1 phase cells were also increased in Cyr61^+^ activated group (*P* < 0.05, Figure 7A,B).

**FIGURE 8:**
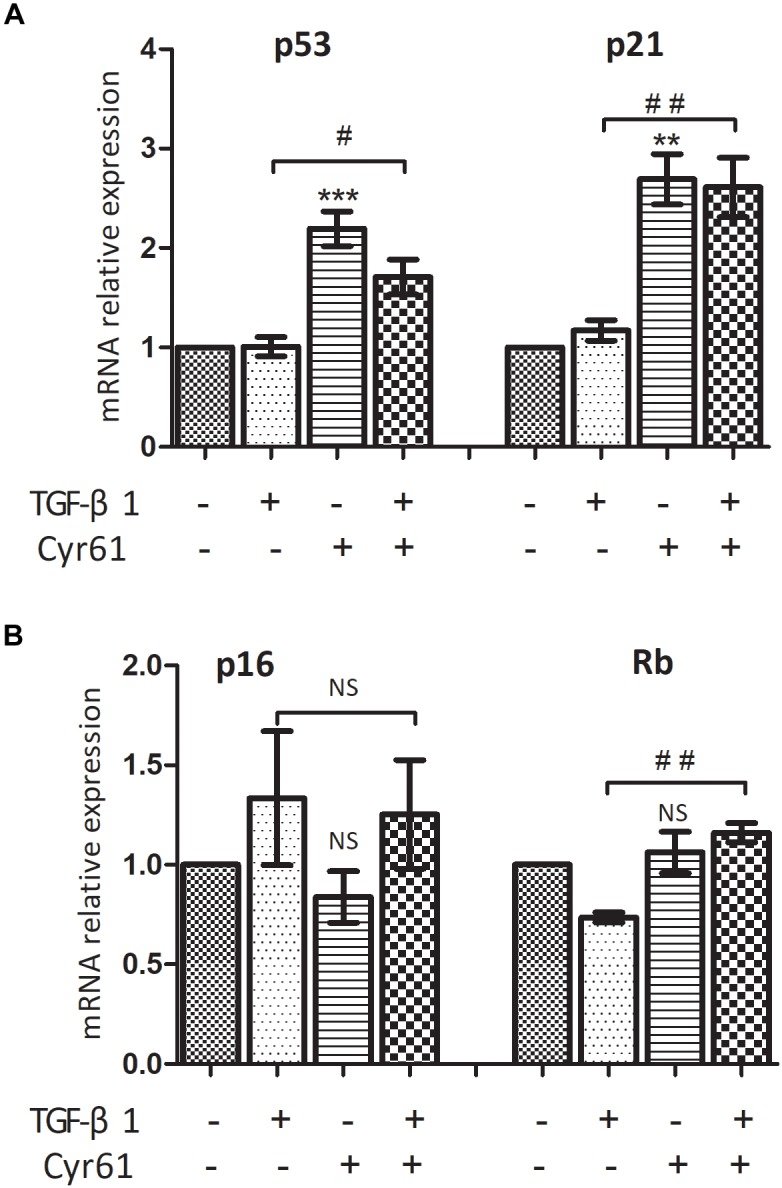
Cyr61 increased the transcription of senescence inducing pathway related factors in renal fibroblasts. These factors (P53, P21, P16, and Rb) were detected by real-time PCR **(A,B)**. NS, no significance; ^∗∗^*P* < 0.01 and ^∗∗∗^*P* < 0.001 vs. control group; ^#^*P* < 0.05 and ^##^*P* < 0.01 vs.activated group.

PCR showed that the transcription of P53 and P21 in Cyr61^+^ group was higher than those of the control group (*P* < 0.01, Figure 8A). Moreover, transcription of P53, P21, and Rb in Cyr61^+^ activated group was more than those of the activated group (all *P* < 0.05, Figure 8A,B). There was no significant difference in the transcription of Rb between Cyr61^+^ group and control group and no difference in p16 among all the groups (all *P* > 0.05, Figure 8A,B).

### Interference With Cyr61 Expression Promoted the Phenotypes of Renal Fibroblasts

**FIGURE 9:**
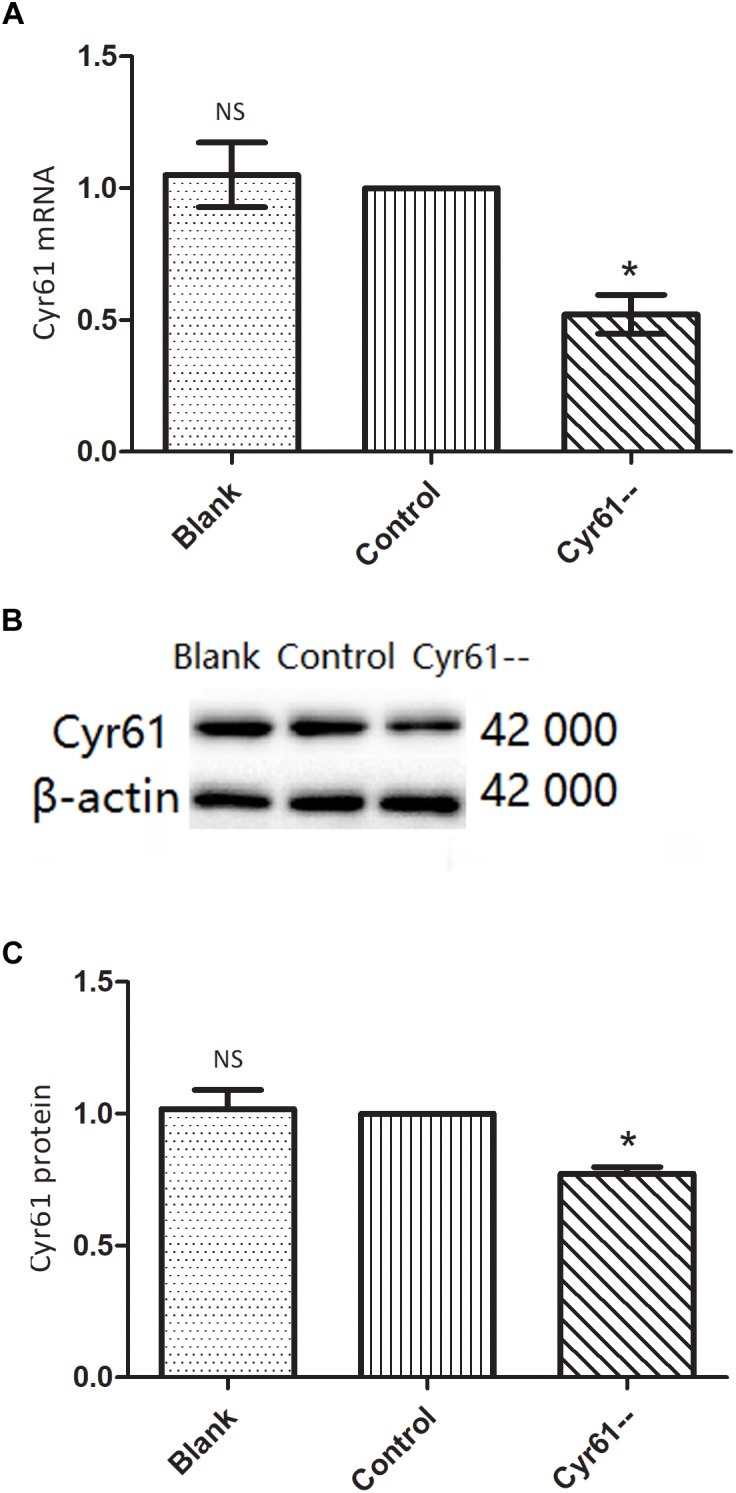
NRK-49F cells with low expression of Cyr61 by plasmids transfection. As a negative contrast experiment, we interfered with Cyr61 expression in NRK-49F cells and grouped cells into blank group, control group (null vector transfection) and Cyr61^--^ group. The transcriptions of Cyr61 were detected by real time PCR **(A)** and the expression of Cyr61 protein was detected by western blotting **(B)**. Relative protein levels based on western blot results **(C)**. NS, no significance, ^∗^*P* < 0.05 vs. Control.

To further verify the function of Cyr61, we also silenced Cyr61 expression in NRK-49F cells and constructed Cyr61 low-expression (Cyr61^--^) group. PCR and western blotting showed that compared with blank group and control plasmid group, Cyr61 mRNA and protein expression were at a low level in Cyr61^--^ group (*P* < 0.05, Figure 9A–C). However, there was no significant difference between blank group and control plasmid group (*P* > 0.05, Figure 9A–C).

**FIGURE 10:**
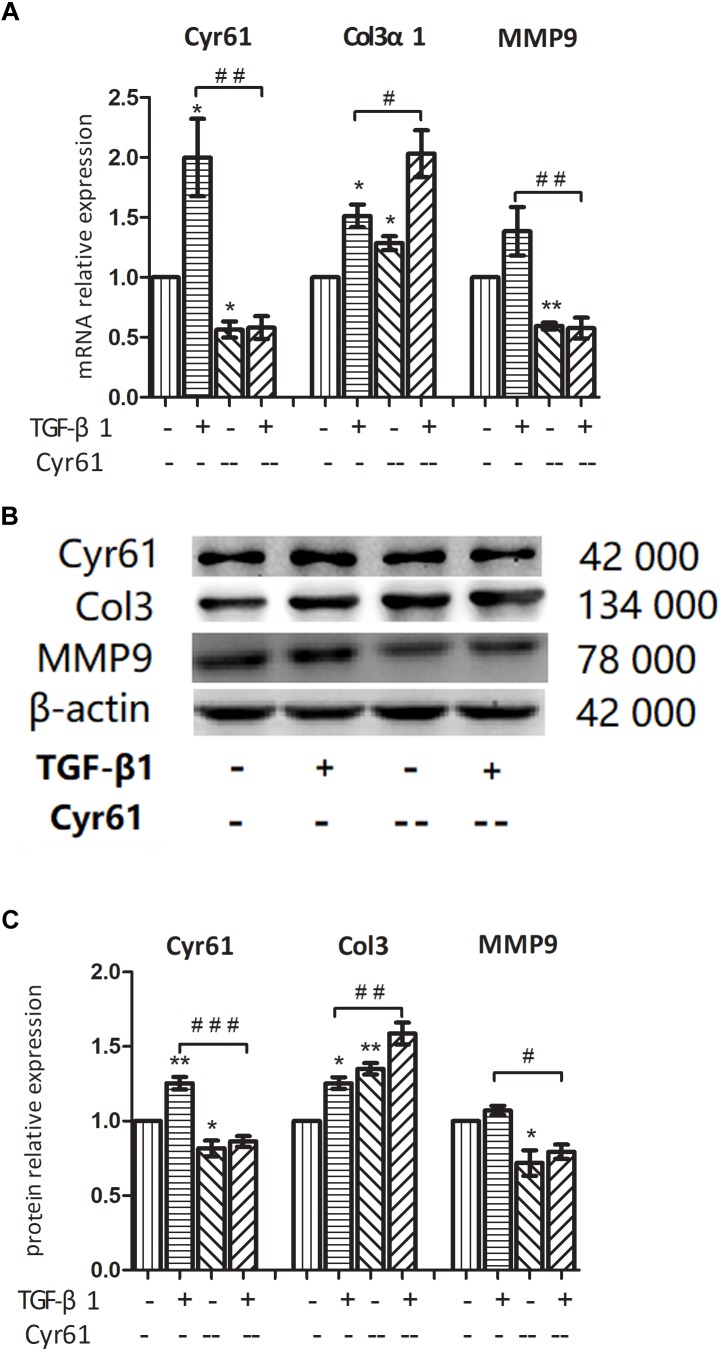
Interference with Cyr61 expression promoted the phenotypes of renal fibroblasts. NRK-49F cells were grouped into four groups: control group, activated group, Cyr61^-^ group and Cyr61^--^ activated group. The expression of Cyr61, Col3α1 and MMP9 were detected by real time PCR **(A)** and western blotting **(B)**. Relative protein levels based on western blot results **(C)**. ^∗^*P* < 0.05 and ^∗∗^*P* < 0.01 vs. control group; ^#^*P* < 0.05, ^##^*P* < 0.01, and ^###^*P* < 0.001 vs. activated group.

On the basis of TGF-β1 activation and Cyr61 knockdown, the cells were divided into the control group, activated group, Cyr61^--^ group, and Cyr61^--^ activated group. PCR and western blotting showed that compared with the control group, the mRNA and protein expression of Cyr61 in Cyr61^--^ group were at low level. The expression of Col3α1 was increased, whereas the expression of MMP9 was decreased (*P* < 0.05, Figure 10A–C).

### Interference With Cyr61 Expression Promoted Cell Proliferation, Promoted Cell Cycle, and Reduced the Transcription of Senescence-Related Factors in Renal Fibroblasts

**FIGURE 11:**
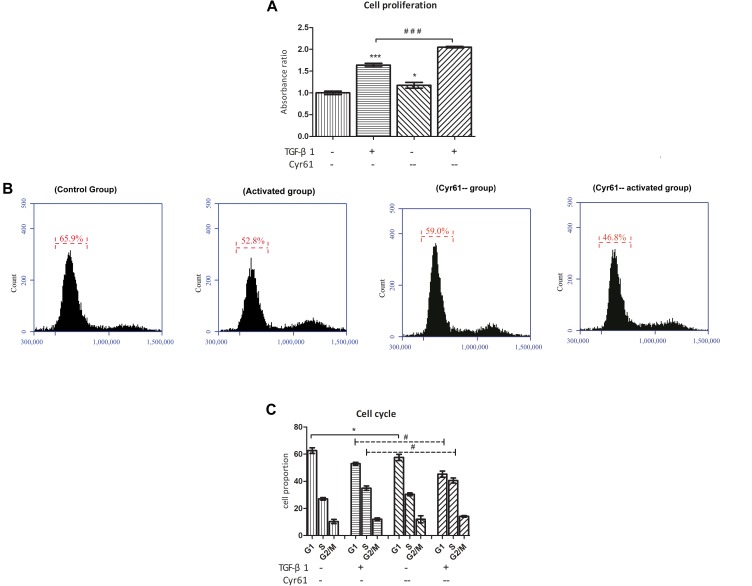
Interference with Cyr61 expression promoted the proliferation and cell cycle progression of renal fibroblasts. The cell viability were assessed by CCK-8 assay **(A)** and the cell cycle were detected by flow cytometry **(B)**. Percentage of all phases based on cell cycle results **(C)**. ^∗^*P* < 0.05 and ^∗∗∗^*P* < 0.001 vs. control group; ^#^*P* < 0.05 and ^###^*P* < 0.001 vs. activated group.

**FIGURE 12:**
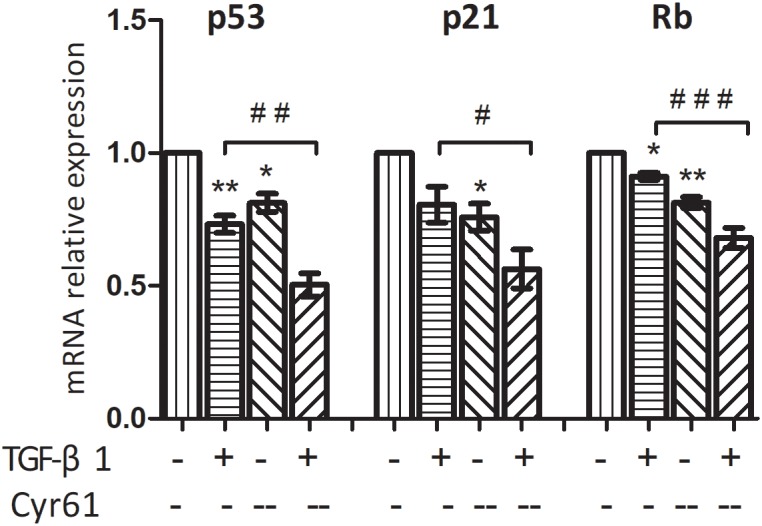
Interference with Cyr61 expression reduced the transcription of senescence related factors in renal fibroblasts. These factors (P53, P21, and Rb) were detected by real time PCR. ^∗^*P* < 0.05 and ^∗∗^*P* < 0.01 vs. control group; ^#^*P* < 0.05, ^##^*P* < 0.01, and ^###^*P* < 0.001 vs. activated group.

CCK-8 results showed that compared with the control group, the cell viability of Cyr61^--^ group was enhanced (*P* < 0.05, Figure 11A). Also, compared with the activated group, the cell proliferation in the Cyr61^--^ activated group was significantly enhanced (*P* < 0.001, Figure 11A). At the same time, the results of flow cytometry showed that compared with the control group, the proportion of fibroblasts in the G1 phase of activated group and also Cyr61^--^ group (all *P* < 0.05, Figure 11B,C) was decreased. Compared with the activated group, the G1 phase cells in Cyr61^--^ activated group were also decreased, and the difference was statistically significant (*P* < 0.05, Figure 11B,C). Further, PCR showed that compared with the control group, the transcription of P53, P21, and Rb was decreased in activated group and Cyr61^--^ group (all *P* < 0.05, Figure 12). Compared with the activated group, the transcription of these factors in the Cyr61^--^ activation group was also significantly reduced (all *P* < 0.05, Figure 12).

## Discussion

Cyr61 is a cysteine-rich secreted protein with heparin binding activity, which is widely found in heart, lung, brain, pancreas, placenta, fetal kidney, and other organs. Cyr61 plays an important role in regulating ECM production, cell differentiation, proliferation, apoptosis, and angiogenesis (Leask and Abraham, 2006). The studies based on the relationship between Cyr61 and organ fibrosis found that Cyr61 was closely related to the physiological state of fibroblasts in various organs, but the effects were not consistent. The study of cutaneous wound healing has found that Cyr61 activated the RAC1-NOX1 complex to induce the generation of reactive oxygen species (ROS) by adhering to integrin α6β1 and HSPGs, thus inducing DNA damage response, p53 activation, and leading to fibroblasts senescence and concomitant expression of antifibrotic genes (Jun and Lau, 2010). A study on liver fibrosis has found that Cyr61 can also induce the transformation of activated fibroblasts into senescent cells, thereby alleviating liver fibrosis (Kim et al., 2013). However, another study on lung fibroblasts has found that Cyr61 affects TGF-β1/SMAD3 signaling pathway and promotes pulmonary fibrosis (Mandal et al., 2010).

In our study, we investigated the expression of Cyr61 in rat kidney tissue. The animal experiments showed that fibrosis was at peak while Cyr61 protein was at the lowest, and fibrosis was decreased while Cyr61 protein was increased. The reduction in renal fibrosis after AKI 2W may be related to organ self-healing ability, which may involve a relatively high expression of Cyr61. In addition, we also comprehended the contradiction between the reduction in fibrosis after AKI 2W and the persistently high level of Scr. Furthermore, we interpreted it that the renal function may be not only associated with interstitial fibrosis but also with impaired renal tubules and interstitial vascular atrophy after IR.

Cysteine-rich protein 61 was found to be predominantly expressed in renal tubular epithelial cells 5 days after IR or 10 days after unilateral ureteral obstruction injury, but it is absent in the fibrotic area (Lai et al., 2013, 2014). Renal fibroblasts are the most important cellular components in the fibrotic area, which can be activated for rapid proliferation and secretion of ECM to achieve repair and even fibrosis. Our bioinformatics analysis found that Cyr61 was decreased in activated renal fibroblasts at 3 days after I/R. However, it is not clear whether the lack of Cyr61 in fibroblasts is associated with renal interstitial fibrosis.

After intraperitoneal injection of anti-Cyr61 antibody in mice with ischemic kidney injury, Lai found that the transcription and expression of collagen were significantly reduced at 14 days and renal fibrosis was alleviated (Lai et al., 2014). Interestingly, these results also showed that the transcription of Col1α1 and Col3α1 was increased at 7 days. It was difficult to identify the role of Cyr61 on renal fibroblasts based on these studies because of the interaction and compensatory effect of various cells *in vivo*.

**FIGURE 13:**
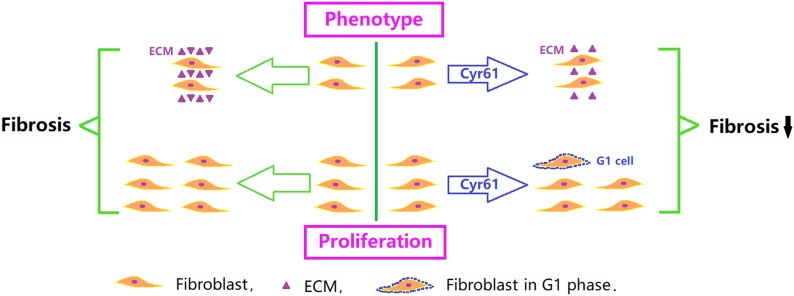
Critical role of Cyr61 on renal fibroblasts and fibrosis. Cyr61 may reduce ECM and inhibit proliferation by arresting fibroblasts in G1 phase, subsequently affecting the process of ischemic renal fibrosis.

To further clarify the role of Cyr61 on renal fibroblasts, we constructed NRK-49F cells with overexpression of Cyr61 (Cyr61^+^) *in vitro* experiments. Our results showed that Cyr61 could significantly reduce the expression of Col1 and Col3 and increase the expression of MMP9 and MMP13. Col1 and Col3 are recognized as ECM structural components. MMP-13 is a highly specific protease capable of degrading insoluble fibrillar collagens, especially Col1. The changes in these three factors all suggested that Cyr61 inhibited the fibrotic phenotype of renal fibroblasts and promoted ECM degradation (Figure 13). However, Cyr61 could also increase the expression of MMP9, and the effect of MMP9 remains controversial. Many groups found that increased activity of MMP9 may have an antifibrotic effect (Cabrera et al., 2007), and an MMP9 inhibitor has been used to construct some renal fibrosis models, while other groups found that MMP9 plays a pro-fibrosis role by promoting macrophage accumulation (Kui Tan et al., 2013). The specific role and mechanism of MMP9 on NRK-49F cells in our experiment need further study.

In the process of fibrosis, activated fibroblasts are mainly reduced in three ways–apoptosis pathway (Wang et al., 2017), recovery of inactivated phenotype (Kisseleva et al., 2012), and aging (Krizhanovsky et al., 2008). The cell senescence pathway not only reduces the number of fibroblasts by stopping growth and immune clearance, but also initiates anti-fibrosis gene programs with senescence-associated secretory phenotype or senescence messaging secretome (SASP/SMS), reducing the synthesis and promoting the degradation of ECM. In the process of cell senescence, the signal transduction pathway mediated by Rb and P53 plays an important role. The non-phosphorylated retinoblastoma protein (Rb) can bind to the E2F transcription factor and shield its transcriptional activation domain, inhibiting the transcription of the enzyme genes (such as DNA polymerase) and inhibiting the cells from phase G1 into S phase (Rayess et al., 2012). In this signaling pathway, P16 can participate in maintaining the non-phosphorylation state of Rb. P53, the core gene product of another senescence pathway, is a stress protein that pauses the G1 restriction point by inducing P21 expression to inhibit the activity of the cell cycle complex cyclinA/E-CDK2 (Sherr and Weber, 2000). The P53 pathway and Rb pathway are not independent, but these interact at multiple levels. For example, P21 can inhibit the phosphorylation of Rb to accelerate senescence. And, Rb can inhibit the P53 degradation mediated by MDM2 (a ubiquitin ligase) (Yap et al., 1999), thus promoting the P53 pathway and causing cell growth stagnation.

In our study, we found that overexpression of Cyr61 significantly reduced proliferation, promoted NRK-49F cells arrest in the G1 phase, and increased transcription of cellular senescence signaling molecules (P53, P21, and Rb). These results indicated that Cyr61 might affect the P53/P21/Rb signaling pathway to promote senescence, thereby inhibiting fibroblasts proliferation (Figure 13). As a negative comparison experiment, this study also constructed a kidney fibroblast model with low expression of Cyr61 by plasmid transfection. The results still support the negative regulation of Cyr61 on renal fibroblasts. Admittedly, renal fibrosis after IR-AKI is involved in cell damage, inflammation, cell activation, remolding, and fibrosis. Our experiments only explored the effect of Cyr61 on renal fibroblasts *in vitro*. The special role of Cyr61 in the whole process of renal fibrosis needs further exploration.

## Conclusion

In conclusion, our results suggested that Cyr61 might not only curb the fibrotic phenotype of fibroblasts, but also inhibit proliferation by promoting fibroblasts arrest in the G1 phase through the P53/P21/Rb interrelated cell senescence pathway, subsequently affecting the process of ischemic renal fibrosis.

## Ethics Statement

This study was carried out in accordance with the recommendations of “International Association of Veterinary Editors guidelines.” The protocol was approved by the “Medical Ethics Committee of Affiliated Hospital of Qingdao University.”

## Author Contributions

HL performed the design, experiments, and thesis writing. LZ performed the animal experiments and experimental guidance. JZ performed the design and experimental guidance. ChL performed the bioinformatics analysis and data analysis. XS, XL, and WJ performed the animal experiments and data analysis. CoL performed the cell experiments and data analysis. YW and LC performed the cell experiments and thesis writing. YX performed the design, experimental guidance, and data analysis.

## Conflict of Interest Statement

The authors declare that the research was conducted in the absence of any commercial or financial relationships that could be construed as a potential conflict of interest.

## Footnotes

1 http://www.ncbi.nlm.nih.gov/geo/
